# Comparing the Effectiveness of Clinicians and Paraprofessionals to Reduce Disparities in Perinatal Depression via the Mothers and Babies Course: Protocol for a Cluster-Randomized Controlled Trial

**DOI:** 10.2196/11624

**Published:** 2018-11-20

**Authors:** Jessica K Jensen, Jody D Ciolino, Alicia Diebold, Melissa Segovia, Aria Degillio, Jesus Solano-Martinez, S Darius Tandon

**Affiliations:** 1 Center for Community Health, Institute for Public Health and Medicine Northwestern University Feinberg School of Medicine Chicago, IL United States; 2 Department of Preventive Medicine Northwestern University Feinberg School of Medicine Chicago, IL United States

**Keywords:** depression, postpartum, pregnancy, randomized controlled trial, community health

## Abstract

**Background:**

Postpartum depression is highly prevalent in low-income women and has significant health and mental health effects on mother and child. Home visiting (HV) programs provide services to large numbers of perinatal women in the United States and are a logical setting for delivering mental health services. Although there are interventions that reduce the risk of developing postpartum depression among low-income women, none have used nonhealth or nonmental health professionals as interventionists.

**Objective:**

This study aimed to outline the protocol of a cluster randomized trial funded by the Patient-Centered Outcomes Research Institute that evaluates whether the Mothers and Babies (MB) group intervention, when led by paraprofessional home visitors, is more efficacious than usual care. It will also examine if MB, when led by home visitors, is not inferior to MB delivered by mental health professionals (MHPs). MB has previously demonstrated efficacy when delivered by MHPs, and pilot work indicated promising results using home visitors to deliver the intervention.

**Methods:**

A cluster randomized trial is being conducted with 38 HV programs. Sixteen HV programs will deliver MB using MHPs, 16 will deliver MB using paraprofessional home visitors, and 6 will deliver usual HV services. The study employs a modified covariate-constrained randomization design at the site level. We anticipate recruiting 933 women aged ≥16 years enrolled in HV programs, who are 33 or more weeks’ gestation and speak either English or Spanish. Women in the 2 intervention arms will receive the 6-session MB group intervention. Baseline, postintervention, 12-week postpartum, and 24-week postpartum assessments will be conducted to assess client outcomes. The primary outcome will be the change in Quick Inventory of Depressive Symptomatology Self-Report 16 scores from baseline to 24-week follow-up. Secondary outcomes associated with core MB content will also be examined. Semistructured interviews will be conducted with home visitors and MHPs who are group facilitators and 90 study participants to gain data on intervention successes and challenges. Analyses will proceed at the participant level. Primary analyses for depressive symptoms score at 24 weeks postpartum will involve a linear mixed model, controlling for baseline symptoms and other covariates, and random effects to account for clustering.

**Results:**

We have recruited 838 women through the end of August 2018. Recruitment will be completed at the end of September 2018.

**Conclusions:**

There is considerable potential to disseminate MB to HV programs throughout the United States. Should our results demonstrate home visitor efficacy when compared with usual care and/ noninferiority between home visitors and MHPs in improving mental health outcomes, no additional financial resources would be required for the existing HV staff to implement MB. Should this study determine that home visitors are less effective than MHPs, we will generate more wide-scale evidence on MB effectiveness when led by MHPs.

**Trial Registration:**

ClinicalTrials.gov NCT02979444; https://clinicaltrials.gov/ct2/show/NCT02979444 (Archived by Webcite at http://www.webcitation.org/archive.php)

**International Registered Report Identifier (IRRID):**

PRR1-10.2196/11624

## Introduction

### Background

Postpartum depression is a serious mental health disorder that poses significant health and mental health risks for mothers and their infants [1]. Research suggests that prevalence rates of postpartum depression are higher among low-income women than among middle- or high-income women [2,3]. There is also consistent evidence that low-income women are less likely to receive mental health services in the perinatal (ie, pregnancy until child’s first birthday) period than their more affluent counterparts due to a variety of factors, including stigma related to mental health service use and lack of access to community-based mental health providers [4,5]. Postpartum depression is a particularly serious problem for low-income women. It is estimated that more than 10% of infants from low-income households have a mother who has major depression and more than 50% have a mother with some depressive symptoms [6]. Postpartum depression also has negative consequences for maternal parenting practices. Compared with women not suffering from postpartum depression, depressed women tend to be less positive, less spontaneous, and less responsive with their infants [7]. Postpartum depression has been linked to developmental delays among infants of depressed mothers, including social interaction difficulties, attachment insecurity, and cognitive impairments [8,9].

Systematic reviews have highlighted an array of efficacious postpartum depression preventive interventions [10]. Among those interventions that have demonstrated efficacy, the majority use health (eg, nurses, midwives) or mental health (eg, psychologists) professionals to deliver individualized or group-based interventions [10]. One exception is the use of peers to deliver peer support via phone [11], although this study was conducted in Canada with predominately white, upper- and middle-class women. As such, there are no interventions led by nonhealth or nonmental health professionals that have demonstrated efficacy in preventing the onset of postpartum depression and reduction of depressive symptoms among low-income women.

Home visiting (HV) programs that provide services to perinatal women are one of the largest avenues through which perinatal women come to the attention of service providers, making HV a unique and viable setting for delivering mental health services. Although professional HV models exist (eg, nurse-family partnership), most HV programs in the United States use paraprofessional home visitors, who lack formal training in the helping professions [12]. Maternal depression is an enormous challenge facing HV programs, with an estimated 10 to 15% of HV clients exhibiting major depressive disorder (MDD) and another 45 to 50% exhibiting subthreshold depressive symptoms [13]. Furthermore, there is consistent evidence that low-income women exhibiting depressive symptoms—including women enrolled in HV programs—do not access mental health treatment in the community [4]. Lack of available mental health professionals (MHPs), stigma in seeking mental health services, and logistical challenges (eg, childcare, transportation) are a few of the barriers low-income women face when seeking mental health services. For those clients who do access services, most perinatal women are likely to receive pharmacological treatments [14], despite the fact that the vast majority of perinatal women prefer nonpharmacologic interventions [5]. HV programs are ideal settings for delivering mental health care to perinatal women because their mission is not stigmatizing and HV programs tend to be trusted entities in the communities they serve [13,15]. Several interventions aimed at treating postpartum depression among women in HV programs have been developed and empirically tested to show efficacy, including an in-home cognitive behavioral therapy (CBT) intervention delivered by licensed masters-level social workers [16,17], a culturally adapted version of interpersonal psychotherapy delivered by masters-level psychiatric nurses [18], and listening visits delivered by home visitors or obstetrician clinic staff focused on empathic listening, collaborative problem solving, and assessment of need for additional mental health treatment [19]. This study was born out of HV programs’ need and desire for a low-cost intervention focused on the prevention of postpartum depression, given the large number of women with subthreshold symptoms at risk for developing MDD.

### Prior Work

Previously, study investigators established the efficacy of a group-based intervention—Mothers and Babies (MB)—in preventing the onset of postpartum depression and reducing depressive symptoms *when led by mental health professionals* in a group setting [20-22]. On the basis of these randomized controlled trials (RCTs), MB is listed as an evidence-based practice in the Health Research and Services Administration registry and is also listed on the Substance Abuse and Mental Health Services Administration Evidence-Based Program Registry [23]. Subsequently, the principal investigator (PI) and colleagues worked closely with HV clients, staff, and other key stakeholders to develop training and implementation protocols to facilitate implementation of the MB group model *by paraprofessional home visitors*. In particular, training protocols and instructor manuals were modified to provide greater clarity on key aspects of MB’s cognitive-behavioral underpinnings. Results from a pilot study with 2 HV programs in Baltimore indicated that women receiving the MB group intervention delivered by paraprofessional home visitors showed improvements in depressive symptoms, suggesting that the MB intervention could be delivered by home visitors instead of MHPs (unpublished data [24]). This project builds on this preliminary work by evaluating the effectiveness of the MB group model when delivered by paraprofessional home visitors.

MB is a 6-session group intervention with content based on 2 key theoretical frameworks—CBT and attachment theory. MB contains 3 modules that align with key CBT elements: pleasant activities, thoughts, and social support and contact with others. Within each module, intervention recipients first are taught to connect each CBT concept with their mood, with subsequent content providing specific skills and techniques to help them cope with stress and depressive symptoms. Attachment theory is woven throughout the curriculum to help intervention participants promote connection with their infants. For example, one way attachment theory is integrated into the pleasant activities module is to highlight that parents can engage in pleasant activities with their child or children which simultaneously can improve a parent’s mood and attachment with their child or children.

### Study Aims

This study is a cluster-randomized controlled trial (C-RCT) in which HV clients receive either (1) MB group delivered by MHPs, (2) MB group delivered by paraprofessional home visitors, or (3) usual HV services. There are 4 specific aims for this study; the first 2 reflect our primary study aims, with the remaining 2 reflecting secondary aims:

Aim 1 (primary aim): Evaluate efficacy of MB delivered by paraprofessional home visitors in comparison with usual care (ie, HV without MB) on patient-reported outcomes, including depressive symptoms, quality of life, parenting practices, engagement in pleasant activities, and relationship with one’s partner.

Aim 2 (primary aim): Assuming efficacy in #1, assess noninferiority (NI) of MB delivered by paraprofessionals versus MHPs.

Aim 3 (secondary aim): Explore patient characteristics as potential covariates and effect modifiers.

Aim 4 (secondary aim): Examine the feasibility and acceptability of MB delivered by paraprofessional home visitors and MHPs.

## Methods

### Study Design and Intervention Delivery

As noted above, this study is a C-RCT with 3 study arms: (1) MB group delivered by MHPs, (2) MB group delivered by paraprofessional home visitors, or (3) usual HV services. This study was approved by the Northwestern University’s institutional review board (IRB). The MB group curriculum has 6 sessions, with each session designed to last 90 to 120 min. The curriculum consists of 3 modules that map onto key components of CBT: pleasant activities, thoughts, and contact with others. The first part of each module teaches participants to understand how a given component influences their mood. Subsequently, participants receive concrete skills related to each module. These skills provide participants with a *toolkit* of skills they can use to improve their mood. We refer to each 6-session MB group as a cohort. Each cohort meets weekly for 6 consecutive weeks at the HV program site, with occasional groups skipping a week due to inclement weather or holidays. Light refreshments are provided at each session. Transportation to the sessions and child care supports are also provided for participants, if necessary. All MB sessions are audio-recorded using a portable device for purposes of examining intervention fidelity. The study design called for random selection of 20% of these audio sessions to be assessed and coded for fidelity. The group facilitator transfers the recordings to Northwestern University’s research team within 24 hours of each individual session using a secure Northwestern University box account. At the end of August 2018, 115 cohorts were completed. Implementation of all prenatal MB cohorts will be completed by October 2018.

### Interventionist Training and Supervision

We have trained 105 paraprofessional (bachelor’s degree or less) home visitors and supervisors from 16 program sites that are using home visitors to deliver the MB intervention. Of the 105 home visitors trained, to date, 33 have delivered the intervention. We have also trained 32 MHPs from the 16 intervention sites using MHPs to deliver MB; 21 of these MHPs have delivered the intervention. MHPs, for the purposes of this study, are masters-level professionals in the areas of child and family studies, psychology, psychiatry, social work, or a related field with a minimum of 5 years’ experience working with families and young children. These MHPs live and work in the states in which they deliver the intervention, and either the participating HV programs or a state professional association (eg, The Illinois Association for Infant Mental Health) recruited them.

The study PI led MB trainings, consisting of 8 to 12 contact hours, for home visitors and MHPs. The PI conducted a total of 19 trainings in the 7 participating states. All the trainings maintained the same contact hours with trainees but were delivered in 3 formats: in-person, webinar, and telephone. HV supervisors from each of the HV programs (irrespective of study arm) also attended the training. The MB training covers the conceptual underpinnings of MB (eg, its cognitive-behavioral framework), a brief history of previous implementation of the MB program with diverse perinatal populations, instruction on the format of the MB instructor manual, and instruction on how to maximize the use of the group format when delivering MB. Training includes discussion of each MB session from start to finish. Training is interactive with opportunities for discussion and modeling communication of material by the PI. Training also involves group activities, where training attendees practice delivering curriculum material and receive extensive feedback on strengths and areas needing improvement from the trainer and other training participants.

Home visitors and MHPs receive phone supervision from the PI the first time they deliver MB. During these supervision sessions, the PI first debriefs the completed MB session and then helps the facilitator plan for the subsequent group session. For home visitors who continue to facilitate groups, the HV program manager assumes the supervisory role—with support from the research team. Along with support from the PI during supervision, MHPs and paraprofessional home visitors can share and receive feedback via the study ListServ, which includes other HV staff, MHPs, HV supervisors, and the research team.

### Recruitment and Informed Consent

Women meeting eligibility criteria for the C-RCT are approached by HV staff who explain the MB intervention and research study. Interested women complete a referral form with the HV staff and are informed that a Northwestern University research assistant (RA) will contact them with more information about the research study. HV staff send the referral forms to the Northwestern research team via email or fax. RAs share responsibility for calling referred women to explain the study in more detail and complete the informed consent process with eligible participants who indicate interest in study participation.

The Northwestern University’s IRB granted a waiver of written documentation of consent, allowing Web-based informed consent via Research Electronic Data Capture (REDCap) [25] or consent via telephone for potential participants without easy access to Web-based resources. If the referred participant meets eligibility criteria and is interested in participating in the study, the RA indicates that a Web link with instruction on completing the baseline assessment via REDCap will be emailed or texted to them.

Participants are informed via informed consent and during all assessments that they may choose to not answer any question at any time for any reason and that not answering questions will not affect their relationship with their HV programs or ability to keep receiving the MB intervention (for those enrolled in the 2 intervention arms). Both the Web-based consent form and the study surveys are available in English and Spanish. Each time a study participant fills out a survey, the survey includes a prompt to the participant asking if they wish to continue participation by completing the next survey.

A waiver of parental permission was granted to waive the signature of parents of children who are participants (pregnant women ≥16 years and <18 years). This study involves minimal risk to the participants by only requiring the administration of Web-based surveys or telephone interviews to collect data. Guidance from the US Department of Health and Human Services Office of Research Protections indicates that individuals aged less than 18 years can consent to study participation without parental consent if the study procedures for which they are consenting are such that they could provide consent outside the research context.

Before beginning group facilitation, all facilitators receive a Web-based informed consent form via REDCap that they must complete before their first group session. In addition to the consent, facilitators are asked to complete a brief demographics questionnaire before facilitating groups and a survey inquiring about supervision support they receive after facilitating each cohort. All consented MHP and home visitors who facilitated an MB cohort are eligible to participate in a semistructured interview. The intervention coordinator approaches them after they have completed facilitation of their last MB cohort. Northwestern University’s IRB approved all recruitment and consent procedures.

### Study Participants

Our recruitment goal for the C-RCT is 933 pregnant women. The 38 HV programs participating in this project enroll clients via referrals from prenatal care clinics; Women, Infants, and Children programs; and other settings working with pregnant women. HV programs implementing MB groups will implement an average of 5 MB cohorts, over the course of the project. Women aged 16 years and older enrolled in HV programs who are 33 or more weeks’ gestation and speak either English or Spanish are eligible for enrollment. Exclusion criteria have been minimized; however, women who have significant cognitive limitations will be excluded as it is not likely they will be able to fully engage in the group-based intervention activities and discussion. Women with high-risk medical and pregnancy conditions will also be excluded as this may preclude women from regularly attending intervention sessions. Women are not excluded based on race and ethnicity or based on demographic characteristics other than the ability to speak English or Spanish.

### Study Sites

HV programs in Illinois, Ohio, Minnesota, Missouri, Michigan, Iowa, and West Virginia that indicated the ability to recruit approximately 40 pregnant women over a 16- to 18-month timeframe were recruited to participate in the study. All HV programs recruit women at high risk for poor pregnancy and parenting outcomes via referrals from prenatal care clinics, community outreach, and current program participation. Moreover, 45 HV programs agreed to participate in the study. We staggered the start of implementation among the programs so that only a subset of the program sites was beginning to implement at one time.

### Randomization

The study employed a modified covariate-constrained randomization [26] design at the HV program level, using unequal (1:3:3; control: MHP delivery of MB: paraprofessional delivery of MB) allocation, with intention to achieve relative balance in a set of prespecified program-level potential covariates. There are 3 variables for which we chose to control imbalance at the study site level at baseline through this approach:

1. Percent non-white clients as reported by the site (treated as a continuous variable).
2. Site yearly client volume (also reported by site and treated as a continuous variable).
3. Population density of the site area (continuous variable).

The covariate-constrained method of randomization allows for efficient balance of multiple covariates at once and is recommended over other methods (ie, simple randomization or matching) for cluster-randomized trials [26]. The general procedure involves:

1. Enumerating a large subset of possible allocation schemes.
2. Evaluating (im)balance for each variable of interest (in this case we have 3) for each possible allocation.
3. If the (im)balance is acceptable according to some prespecified criterion, then we save this scheme in a smaller subset of potential allocations for implementation.
4. Of those that meet acceptable levels of imbalance, we randomly select 1 allocation for use in this study.

We chose the *P* value corresponding to the Kruskal-Wallis test as our criterion for “balance” in step #3 above. If the *P* value for each of the 3 variables is larger than .30 for a given simulated allocation scheme, that particular allocation is deemed “acceptable.” This criterion is adapted from the “Minimal Sufficient Balance” principle from Zhao et al [27] in the individual sequential randomization literature.

Randomization occurred in 3 waves for logistical purposes. The first wave included 14 sites (2 control, 6 mental health professional, 6 HV), the second included 19 sites (4 control, 7 mental health professional, 8 HV), allocation ratio was slightly off in this wave to account for dropout sites), and the third included 12 sites (1 control, 6 mental health professional, 5 HV). Thus, we randomized a total of 45 sites in 3 waves.

After randomization, 7 programs dropped out, thereby yielding a total of 38 active study sites; 6 of these sites removed themselves before implementing the intervention and 1 after beginning implementation and enrolling participants. Data collected from all study participants will be used in the analysis. Among the 38 active study sites, 16 HV programs are receiving the MB intervention delivered by MHPs, 16 are receiving MB intervention delivered by HV paraprofessionals, and 6 programs serve as control sites and are not implementing the MB intervention.

As the study aimed to assess both (1) efficacy of MB delivered by HV in comparison with control and (2) NI of MB when delivered by HV compared with MHPs, sample size considerations and allocation ratio accounted for each. We chose unequal allocation with fewer women in the control arm so that we could first assess efficacy of the HV model versus control. To show efficacy—a significant and meaningful difference between the 2 arms—sample size requirements were not as large as in the NI setting. The NI aim requires larger numbers of women in the 2 active intervention arms; thus, we chose to enroll threefold the number of women in the 2 active intervention arms to ensure maximal efficiency for NI analyses.

### Data Collection Procedures and Study Assessments

The study includes 4 data collection time points—baseline, immediately post-intervention (or 8 weeks after the baseline for control participants), 12 weeks postpartum, and 24 weeks postpartum. Participants will receive US $20 remuneration after completing the baseline, 12-week, and 24-week assessments for a total of US $60. Baseline and follow-up data will be collected and managed using REDCap. Baseline data will be collected within 2 to 3 weeks of establishing client eligibility and participation agreement. Women who do not complete the Web-based baseline assessment in this timeframe will be contacted by phone by the RA to complete the assessment by phone, ensuring that baseline data are collected before the first MB group.

Table 1 describes the study’s outcome indicators, measures, and data collection time points. This study’s primary outcome is reduction in depressive symptoms with several secondary outcomes (eg, behavioral activation, mood regulation) that are closely linked with MB content. For our primary outcome of depressive symptoms, continuous higher scores on the Quick Inventory of Depressive Symptomatology Self-Report 16 (QIDS-SR16) [28] indicate greater depressive symptomatology. For our secondary outcome of major depressive episodes, endorsing 5 or more items on the Maternal Mood Scale and interference with current life activities indicates a possible major depressive episode. For our secondary outcomes of behavioral activation, pleasant activities, mood regulation, social support, decentering, relationships with one’s partner, and subjective well-being, increased scores over time indicate improvement. For our secondary outcome of perceived stress, decreased scores over time indicate improvement. For the secondary outcome of responsive and reactive parenting, higher scores on the Parental Cognitions and Conduct toward the Infant Scale [29] subscales indicate greater self-efficacy, hostile-reactive parenting, perceived parental impact, and parental overprotection over time.

We are collecting data on MB acceptability via 3 data modalities. First, we are conducting brief semistructured interviews with 90 intervention participants—45 who received MB led by MHPs and 45 who received MB led by paraprofessional home visitors. Second, we are conducting brief semistructured interviews with all home visitors and MHPs who deliver MB. Third, all participants will complete brief paper-and-pencil checklists immediately after receiving an MB session. Group facilitators will collect these checklists. We ask each intervention participant to rate each session using 3 questions used in previous MB studies: “how much did you enjoy today’s group session?”, “how well did you understand what we talked about during today’s group session?”, and “how often do you think you will use the skills and information that you were given during today’s group session?”

To assess feasibility of MB delivered by MHPs and home visitors, we are collecting data on (1) number of completed intervention sessions (dosage) and (2) fidelity of intervention implementation. Completed intervention sessions and participant attendance are documented by MHPs and home visitors delivering MB on a form created for use in this study. Fidelity of intervention implementation is being assessed by reviews of audiotaped sessions. All MB sessions will be audiotaped, and a random sample of 20% will be reviewed for protocol adherence by 2 trained coders performing independent ratings of fidelity.

**Table 1:** Study outcome indicators, measures, scoring, and data collection time points.

Outcome indicator		Measure	Scoring range	Scoring interpretation	Baseline	Postintervention	12 weeks postpartum	24 weeks postpartum
**Primary outcome**								
	Depressive symptoms	Quick Inventory of Depressive Symptomatology Self-Report 16 (QIDS-SR16) [28]	0-27	Higher score: greater depressive symptomatology	✓	✓	✓	✓
**Secondary outcomes**								
	Depressive symptoms	Edinburgh Postpartum Depression Scale [30]	0-30	>10: possible depression	✓			
	Major depressive episodes	Maternal Mood Screener [31]	0-9	≥5 symptoms and interference with current life activities: possible major depressive episode	✓		✓	✓
	Behavioral activation	Behavioral Activation Depression Scale [32]	0-54	Higher score: greater behavioral activation	✓		✓	✓
	Pleasant activities	Pleasant Activities Schedule [33]	0-44	Higher score: greater frequency and enjoyment of activities	✓		✓	✓
	Mood regulation	Negative Mood Regulation Scale [34]	30-150	Higher score: greater expectancies for negative mood regulation	✓		✓	✓
	Social support	Medical Outcomes Study (MOS) Social Support Survey [35]	1-5	Higher mean score: greater perceptions of social support	✓		✓	✓
	Decentering	Experiences Questionnaire [36]	11-55	Higher score: greater decentering and rumination	✓		✓	✓
	Relationship with partner	Dyadic Adjustment Scale[37]	7-43	Higher score: greater relationship satisfaction	✓		✓	✓
	Responsive and reactive parenting	Parental Cognitions and Conduct toward the Infant Scale [29]	0-10	Higher mean scores: greater self-efficacy, hostile-reactive parenting, perceived parental impact, parental overprotection			✓	✓
	Subjective well-being	Flourishing Scale [38]	8-56	Higher score: greater psychological resources and strengths	✓		✓	✓
	Perceived stress	4-item Perceived Stress Scale [39]	0-16	Higher score: greater perceived stress	✓		✓	✓

#### Retention Strategies

Recruitment procedures emphasize the importance of participating in all MB sessions and remaining in the study through the 24-week postpartum assessment. The research team obtains ample tracking information at baseline, which includes the participants’ name, email address, home and cell phone numbers, mailing address, HV site, and secondary contacts indicated by the participant. The research team updates contact information and each participant’s preferred mode of communication (eg, phone, text, Facebook) at each follow-up assessment. We allow participants without easy access to the internet and those less comfortable completing surveys electronically to complete follow-up surveys by phone. We conduct intensive follow-up with participants throughout the study via monthly communication from the RAs using the participant’s preferred modes of contact. We follow all study participants through the 24-week postpartum assessment regardless of their attendance at intervention sessions or completion of previous assessments.

#### Data Monitoring Plan

The intervention coordinator refers any study participant who endorses thoughts of self-harm on the QIDS-SR16, Edinburgh Postnatal Depression Scale, or Maternal Mood Screener to the HV program supervisor. The supervisor uses his or her agency’s protocol to make a determination of the necessary action needed to ensure the safety of the study participant. The research team notifies the PI and research project manager immediately of any such referrals. In addition, should a participant indicate experience of severe depressive symptoms upon completion of a depression scale assessment, study staff notify the supervisor at the HV program to provide appropriate referrals for their client’s mental health treatment linkage. In addition to following up with the HV supervisor, RAs also follow-up with study participants to ensure they are not in immediate danger of harming themselves and provide a list of resources to the participant. The statistical team, in collaboration with the rest of the study team, developed a series of data status and quality reports via an automated task, which study staff review multiple times per week. They include participant status, missing survey and overall data, mood assessment summaries, and intervention adherence reports.

### Statistical Analysis

#### Data Management

Study data are collected and managed using REDCap, a secure Web-based application designed to support data capture for research studies, hosted and supported by Northwestern University’s Clinical and Translational Science Institute. Web-based survey data completed by participants are directly linked to the REDCap project. Data are periodically exported to an SAS database that is only accessible to study personnel on a password-protected shared project drive. The research study coordinator, the biostatistician, and the statistical analyst conduct periodic audits of the data to ensure accuracy over the course of the project.

All data records are identified using an identification number only and do not contain other personally identifying information. All forms containing personal identifying information, including a master list linking names with identification numbers, consent forms, and receipts for subject remuneration payments, are maintained in a file cabinet that is locked at all times and on a password-protected computer, with access only by the study team. Identifying information (eg, personal and

contact information) is kept separate from the other data. All subject information collected is kept in secure, password-protected files on Northwestern University’s servers with access restricted to authorized personnel only. Audio-recordings of MB group sessions will be stored as password-protected electronic files on a secure computer. Once the study is completed, including data coding and qualitative analysis, all audio-recordings will be erased. We will inform participants that coded data will be deidentified.

#### Outcomes

Primary outcome of interest for Aims #1 to #3 will be the QIDS-SR16 score as determined by participant self-report at 24 weeks postpartum. We will control for baseline QIDS-SR16 score and treat this measure as a continuous variable. It ranges from 0 to 27 points, where higher scores signify increased depressive symptoms. QIDS-SR16 translates into depressive categories such that a score of less than 5 points indicates no depression, a score ranging from 6 to 10 indicates mild depression, 11 to 15 signifies moderate depression, 16 to 20 indicates severe depression, and anything above 20 would be labeled as very severe [28]. As a result, we deem a 5-unit change or difference in score to be meaningful (as a jump in 5 points would result in an increase in depression severity tier for any individual patient). Secondary outcomes will address key components of the MB intervention. They include incidence of major clinical depression, behavioral activation, engagement in pleasant activities, mood regulation, social support, decentering, perceived stress, responsive parenting, relationship with partner, and subjective well-being.

#### Statistical Methods

Descriptive statistics will summarize baseline characteristics (both site-level and participant-level) overall and by arm. As appropriate, mean (SD) (or median [inner quartile range] will be used in cases of skewed or nonnormal empirical distributions) and frequency (proportions) will summarize continuous and categorical data, respectively. Analyses will employ normal theory methodology as appropriate, and in cases of violations of assumptions, transformations and nonparametric analyses may be utilized. We used SAS version 9.4 (SAS Institute Inc.; Cary, NC; 2012) [40] to perform randomization, and we plan to use both SAS (version 9.4) and R (version 3.4.1 or higher) [41] for final analyses and reporting. We used the Power **a**nd Sample Size (PASS) Software (NCSS, LLC; Kaysville, Utah; 2011 )[42] for all power and sample size calculations.

**Figure 1:**
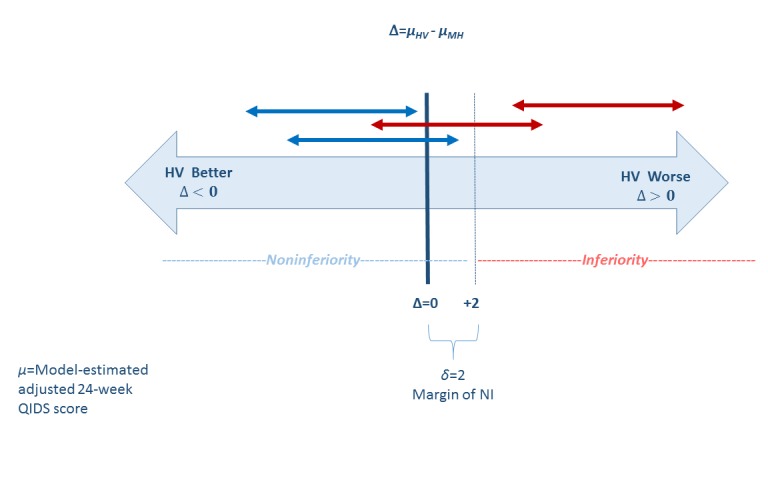
Margin of noninferiority (NI). HV: home visiting; QIDS: Quick Inventory of Depressive Symptomatology.

Analyses will proceed at the participant level. Primary analyses for QIDS-SR16 score at 24 weeks postpartum will involve a linear mixed model for continuous outcome with independent variables of baseline QIDS-SR16, study arm (3-level factor), and site-level baseline covariates used in randomization algorithm. We plan to account for clustering effects via inclusion of a random site effect, which will allow for distinction of between- and within-site variance. Intracluster correlation coefficients (ICCs) will be estimated via variance components estimates. Intervention effect will first be evaluated via the adjusted Wald type III test for significant study arm effect at the 5% level of significance. If arm is significant at the 5% level, analyses evaluating the superiority (Aim #1) of HV-led intervention versus control will proceed with Tukey correction for multiple pairwise hypothesis tests. Assuming the Tukey-adjusted *P* value for this comparison falls below the 5% level of significance in favor of the HV-led arm, we will further assess the NI (Aim #2) via pairwise comparison (using Tukey correction) of adjusted 24-month QIDS-SR16 in home visitor–led versus MHP-led arms. Figure 1 depicts the margin and zone of NI for this comparison.

The double-sided arrows represent 95% CI for the model-estimated adjusted mean 24-week difference in QIDS-SR16 score between arms. Note that higher score signifies more depressive symptoms; thus, if the estimated difference (HV minus MHP score) is larger than zero, the HV arm has on average worse depressive symptoms. As the prespecified margin of NI is 2 units on the QIDS-SR16 scale, we will claim NI if the upper limit of the adjusted 95% CI for the paraprofessional home visitor–led arm minus the MHP-led arm comparison remains below 2. The red arrows indicate scenarios in which we cannot claim NI, and the blue arrows indicate scenarios in which the criterion for NI is met.

Using the same analytic approach, we will add covariate-by-arm interaction terms in the aforementioned model to explore whether effectiveness of intervention varies by patient characteristics (Aim #3). The covariates to be explored include race and ethnicity, first-time mother, and geographic type of HV program (urban, suburban, or rural). These analyses are more exploratory in nature, and thus power and sample size considerations do not focus on interaction effects. As a result, we anticipate evaluating interaction effects at the 10% level of significance without adjustment for multiple hypothesis tests. Qualitative data analysis methods will evaluate feasibility and acceptability of MB in each setting (Aim #4), and the details of these analyses will be specified elsewhere.

Additional analyses surrounding Aims #1 to #3 will employ longitudinal methods, utilizing all study data at all time points (ie, inclusion of a fixed study time point effect). Secondary outcomes will all be analyzed in this fashion with the exception being binary response variables (eg, depression onset). In these cases, models will involve appropriate link and distributional assumptions (logit and binomial, respectively). For the participants receiving active intervention (either paraprofessional home visitor–led or MHP-led MB), we will examine a “dose” variable in relation to outcomes. This dose variable will be defined as the number of sessions (of 6 possible) attended for an individual participant.

We plan to conduct analyses on the modified intent-to-treat dataset whereby all participants randomized with data at the 24-week postpartum time point will be analyzed according to the arm to which they were allocated. We will further perform a sensitivity analysis on the “as treated” dataset. Those in either active intervention arm will be considered “treated” if they attend at least 4 of the 6 sessions required for the MB course.

Power and sample size considerations allowed for some missing data and, as specified in our approved research plan, we have allowed for entire clusters to drop out of each arm; however, in the event of large amounts of missing data (ie, more than 15%), we will explore multiple imputation analyses. We will examine rates of missing data for all variables and determine whether the rates vary by participant characteristics, HV program location, or intervention arm. These summarizations will inform potential biases resulting from missing data. The mixed effects models planned for analyses are generally robust for unbalanced data across study time points. If multiple imputation methods are merited, we will impute at least five datasets to generate an estimated average intervention effect. These analyses will again serve as sensitivity analyses to the previously outlined analyses.

#### Power and Sample Size Considerations

As in any C-RCT, power and sample size considerations depend heavily on ICC estimates. In general, it is recommended that these calculations account for anticipated ICC, as failure to do so in the design phase leads to an increase in type II error (ie, result in underpowered studies) [43,44]. Without previous knowledge of ICC(s) in this population with respect to our primary outcome, we explored a range of ICCs (0.001 to 0.05). Power calculations assume a SD in primary outcome of approximately 6 points (on the QIDS-SR16), with a meaningful difference corresponding to 5 points on average across arms.

**Table 2:** Required sample size for 90% power in noninferiority aim.

ICC^a^	Sites, n		Study participants per site for analyses, n	Study participants recruited per site (allowing for 15% attrition), n
	HV^b^	MHP^c^		
.001	16	16	17	20
.01	16	16	20	23
.02	16	16	25	29
.03	16	16	33	39
.04	16	16	49	58
.05	16	16	97	114
.001	15	15	19	22
.01	15	15	22	26
.02	15	15	28	33
.03	15	15	38	45
.04	15	15	62	73
.05	15	15	168	198

^a^ICC: intracluster correlation coefficient.

^b^HV: home visiting.

^c^MHP: mental health professional.

Thus, for the superiority aim, we calculated power based on the average ability to detect at least a 5-point mean difference between control sites and paraprofessional home visitor–led sites. Power calculations assumed a 5%/3=1.7% level of significance to account for 3 pairwise comparisons. This is an approach that we deem conservative as it mirrors the Bonferroni correction for multiple hypothesis tests.

We have 38 active sites: 6 controls, 16 paraprofessional home visitor–led sites, and 16 MHP-led sites. We plan to recruit an average of 27 participants per site, allowing for up to 15% attrition (ie, 23 participants on average for analyses). These assumptions allow for more than 95% power to detect a mean difference of 5 points in QIDS-SR16 across arms for an ICC of 0.01. Even if 1 of the 6 sites drops out, and if ICC is as large as 0.05 (which we deem unlikely), we still anticipate over 85% power for analyses addressing the efficacy aim (Aim #1).

Power for the NI aim will require ability to detect a smaller (margin of NI of 2 points) mean difference across arms. We anticipate over 90% power to detect a margin of NI of 2 points on the QIDS-SR16 scale if we assume an ICC of 0.01 and 16 sites in each of the intervention arms with 23 participants for analyses, on average, per site. This allows for up to 15% attrition overall. We anticipate some sites to be over or underperforming, and thus, unequal representation per site is inevitable. Our hope is that the precautions taken with respect to the randomization algorithm that attempts to control imbalance in yearly volume and population density will offset biases created by over/underrepresentation for participants at specific sites. Although we do not anticipate ICC to be larger than 0.01 [43], we present the required sample size per site in Table 2 to ensure 90% power under the same assumptions for all scenarios explored. We also present sample size requirements in the event that one of the active sites drops out in each of the intervention arms (ie, 15 sites per arm). Notice that if ICC is larger than 0.02 (which we are not anticipating, although it remains possible), our projected sample size does not allow for 90% power in this case. As these assumptions are all rather conservative, we argue adequate power for detection of both superiority and NI. Power calculations for assessment of heterogeneity of intervention effects depending on participant characteristics (Aim #3) require even more assumptions for which we have little information. Thus, we do not necessarily anticipate power to detect specific effects within subgroups or power to detect interaction effects, but we plan to use the analyses outlined here to explore these effects.

## Results

This study is in progress. Recruitment for this C-RCT commenced in January 2017, and we anticipate enrollment will continue through September 2018. Through the end of August 2018, we have enrolled 838 women into the study. We have completed qualitative interviews with 5 home visitors and 6 MHPs who have delivered MB cohorts and who will not be facilitating future cohorts, and 55 participants who have received the intervention.

## Discussion

### Comparison With Prior Work and Future Possibilities

This study integrates a low-cost intervention into HV programs, some of which are being infused with new federal funding through the Affordable Care Act. These HV programs serve large numbers of perinatal women at risk for major depression who are often overlooked by the existing mental health services. Should we find that women receiving MB from paraprofessional home visitors exhibit (1) better mental health outcomes than women receiving usual care and (2) similar improvements to women receiving MB from MHPs, there is considerable potential to expand this intervention to HV programs across the country. This is feasible as home visitors are already employed at the setting in which the intervention occurs, thus minimizing the need to procure additional, potentially costly, resources. No previous studies have demonstrated efficacy in preventing the onset of postpartum depression among low-income women using interventions led by nonhealth professionals or non-MHPs. As such, this study will also advance the field of postpartum depression prevention.

This study also allows for the delivery of mental health services outside the public mental health system. The difficulties of paying for prevention and early intervention are well documented, as most health plans require a diagnostic code for billing and do not reimburse for prevention of mental illness including depression. The novel integration of a depression prevention intervention into HV programs provides a potential avenue for delivering depression prevention to women at increased risk for developing major depression in the postpartum period.

### Study Limitations

The study has some limitations that need to be recognized. We are collaborating with HV programs in 7 states in the Midwest. As these sites only represent a subset of HV programs across the country, study results may not be generalizable to all HV programs nationwide. Moreover, HV programs in this study primarily used the Healthy Families America or Parents as Teachers model, thereby potentially limiting generalizability to HV programs using other HV models. Our study is also limited in its ability to examine mediating effects on our primary outcome of depressive symptoms. Although this study represents one of the largest studies to date to examine the effects of a postpartum depression preventive intervention, we did not power the study to formally test for the mediating role of our secondary outcomes—for example, behavioral activation, social support—on depressive symptoms. As with any study, the sample size considerations were based on multiple assumptions (eg, ICC, SD), and if these assumptions are incorrect, the required sample size may be over or underestimated. Finally, multiple sites dropped out as previously indicated; however, we accounted for this potential dropout (both at the site and participant level) in our sample size considerations.

### Conclusions

Despite these limitations, we feel that this study is likely to increase patient engagement in HV programs. As such, study participants will not only see improvements in their own mental health, but are likely to experience greater benefit from the services and supports provided by HV programs aimed at improving positive parenting behaviors, increasing linkages with prenatal and postpartum care, and increasing the use of child preventive health care services (eg, timely well-child visits). Thus, we believe that this study has important implications for improving maternal and child health in multiple domains during the perinatal period.

## Appendix

Multimedia Appendix 1 Peer-review report.

## Abbreviations

CBT: cognitive behavioral therapy
C-RCT: cluster-randomized controlled trial
HV: home visiting
ICC: intracluster correlation coefficient
IRB: institutional review board
MB: Mothers and Babies
MDD: major depressive disorder
MHP: mental health professional
NI: noninferiority
PI: principal investigator
QIDS-SR16: Quick Inventory of Depressive Symptomatology Self-Report 16
RA: research assistant
RCT: randomized controlled trial
REDCap: Research Electronic Data Capture

## References

[1] Gaynes BN, Gavin N, Meltzer-Brody S, Lohr KN, Swinson T, Gartlehner G, Brody S, Miller WC (2005). Perinatal depression: prevalence, screening accuracy, and screening outcomes. Evid Rep Technol Assess (Summ).

[2] O'hara MW, Swain AM (2009). Rates and risk of postpartum depression—a meta-analysis. Int Rev Psychiatry.

[3] Rich-Edwards JW, Kleinman K, Abrams A, Harlow BL, McLaughlin TJ, Joffe H, Gillman MW (2006). Sociodemographic predictors of antenatal and postpartum depressive symptoms among women in a medical group practice. J Epidemiol Community Health.

[4] Abrams LS, Dornig K, Curran L (2009). Barriers to service use for postpartum depression symptoms among low-income ethnic minority mothers in the United States. Qual Health Res.

[5] Leis JA, Mendelson T, Perry DF, Tandon SD (2011). Perceptions of mental health services among low-income, perinatal African-American women. Womens Health Issues.

[6] Vericker T, Macomber J, Golden O The Urban Institute.

[7] van Doesum KT, Hosman CM, Riksen-Walraven JM, Hoefnagels C (2007). Correlates of depressed mothers' sensitivity toward their infants: the role of maternal, child, and contextual characteristics. J Am Acad Child Adolesc Psychiatry.

[8] Grace SL, Evindar A, Stewart DE (2003). The effect of postpartum depression on child cognitive development and behavior: a review and critical analysis of the literature. Arch Womens Ment Health.

[9] Sohr-Preston SL, Scaramella LV (2006). Implications of timing of maternal depressive symptoms for early cognitive and language development. Clin Child Fam Psychol Rev.

[10] Dennis C, Dowswell T (2013). Psychosocial and psychological interventions for preventing postpartum depression. Cochrane Database Syst Rev.

[11] Dennis CL, Hodnett E, Kenton L, Weston J, Zupancic J, Stewart DE, Kiss A (2009). Effect of peer support on prevention of postnatal depression among high risk women: multisite randomised controlled trial. BMJ.

[12] Olds DL, Robinson J, O'Brien R, Luckey DW, Pettitt LM, Henderson CR, Ng RK, Sheff KL, Korfmacher J, Hiatt S, Talmi A (2002). Home visiting by paraprofessionals and by nurses: a randomized, controlled trial. Pediatrics.

[13] Ammerman RT, Putnam FW, Bosse NR, Teeters AR, Van Ginkel JB (2010). Maternal depression in home visitation: a systematic review. Aggress Violent Behav.

[14] Huybrechts KF, Palmsten K, Mogun H, Kowal M, Avorn J, Setoguchi-Iwata S, Hernández-Díaz S (2013). National trends in antidepressant medication treatment among publicly insured pregnant women. Gen Hosp Psychiatry.

[15] Paris R, Dubus N (2005). Staying connected while nurturing an infant: a challenge of new motherhood. Fam Relat.

[16] Ammerman RT, Putnam FW, Stevens J, Bosse NR, Short JA, Bodley AL, Van Ginkel JB (2011). An open trial of in-home CBT for depressed mothers in home visitation. Matern Child Health J.

[17] Ammerman RT, Putnam FW, Altaye M, Stevens J, Teeters AR, Van Ginkel JB (2013). A clinical trial of in-home CBT for depressed mothers in home visitation. Behav Ther.

[18] Beeber LS, Holditch-Davis D, Perreira K, Schwartz TA, Lewis V, Blanchard H, Canuso R, Goldman BD (2010). Short-term in-home intervention reduces depressive symptoms in early head start Latina mothers of infants and toddlers. Res Nurs Health.

[19] Segre LS, Brock RL, O'Hara MW (2015). Depression treatment for impoverished mothers by point-of-care providers: a randomized controlled trial. J Consult Clin Psychol.

[20] Tandon SD, Leis JA, Mendelson T, Perry DF, Kemp K (2014). Six-month outcomes from a randomized controlled trial to prevent perinatal depression in low-income home visiting clients. Matern Child Health J.

[21] Tandon SD, Perry DF, Mendelson T, Kemp K, Leis JA (2011). Preventing perinatal depression in low-income home visiting clients: a randomized controlled trial. J Consult Clin Psychol.

[22] McFarlane E, Burrell L, Duggan A, Tandon D (2017). Outcomes of a randomized trial of a cognitive behavioral enhancement to address maternal distress in home visited mothers. Matern Child Health J.

[23] Substance Abuse and Mental Health Services Administration (SAMHSA) (2018). National registry of evidence-based programs and practices.

[24] Tandon S, Perry D, Mendelson T, Kemp K (2018). Results from a pilot study using paraprofessional home visitors to deliver a postpartum depression preventive intervention. Unpublished data.

[25] Harris PA, Taylor R, Thielke R, Payne J, Gonzalez N, Conde JG (2009). Research electronic data capture (REDCap)--a metadata-driven methodology and workflow process for providing translational research informatics support. J Biomed Inform.

[26] Ivers NM, Halperin IJ, Barnsley J, Grimshaw JM, Shah BR, Tu K, Upshur R, Zwarenstein M (2012). Allocation techniques for balance at baseline in cluster randomized trials: a methodological review. Trials.

[27] Zhao W, Hill MD, Palesch Y (2015). Minimal sufficient balance-a new strategy to balance baseline covariates and preserve randomness of treatment allocation. Stat Methods Med Res.

[28] Rush AJ, Trivedi MH, Ibrahim HM, Carmody TJ, Arnow B, Klein DN, Markowitz JC, Ninan PT, Kornstein S, Manber R, Thase ME, Kocsis JH, Keller MB (2003). The 16-Item Quick Inventory of Depressive Symptomatology (QIDS), clinician rating (QIDS-C), and self-report (QIDS-SR): a psychometric evaluation in patients with chronic major depression. Biol Psychiatry.

[29] Boivin M, Pérusse D, Dionne G, Saysset V, Zoccolillo M, Tarabulsy GM, Tremblay N, Tremblay RE (2005). The genetic-environmental etiology of parents' perceptions and self-assessed behaviours toward their 5-month-old infants in a large twin and singleton sample. J Child Psychol Psychiatry.

[30] Cox JL, Holden JM, Sagovsky R (1987). Detection of postnatal depression. Development of the 10-item Edinburgh Postnatal Depression Scale. Br J Psychiatry.

[31] Vázquez FL, Muñoz RF, Blanco V, López M (2008). Validation of Muñoz's Mood Screener in a nonclinical Spanish population. Eur J Psychol Assess.

[32] Kanter JW, Mulick PS, Busch AM, Berlin KS, Martell CR (2006). The Behavioral Activation for Depression Scale (BADS): psychometric properties and factor structure. J Psychopathol Behav Assess.

[33] MacPhillamy DJ, Lewinsohn PM (1982). The pleasant events schedule: studies on reliability, validity, and scale intercorrelation. J Consult Clin Psychol.

[34] Catanzaro SJ, Mearns J (1990). Measuring generalized expectancies for negative mood regulation: initial scale development and implications. J Pers Assess.

[35] Sherbourne CD, Stewart AL (1991). The MOS social support survey. Soc Sci Med.

[36] Teasdale JD, Moore RG, Hayhurst H, Pope M, Williams S, Segal ZV (2002). Metacognitive awareness and prevention of relapse in depression: empirical evidence. J Consult Clin Psychol.

[37] Crane DR, Middleton KC, Bean RA (2000). Establishing criterion scores for the Kansas Marital Satisfaction Scale and the Revised Dyadic Adjustment Scale. Am J Fam Ther.

[38] Diener E, Wirtz D, Tov W, Kim-Prieto C, Choi D, Oishi S, Biswas-Diener R (2009). New well-being measures: short scales to assess flourishing and positive and negative feelings. Soc Indic Res.

[39] Cohen S, Hoberman HM (1983). Positive events and social supports as buffers of life change stress. J Appl Social Pyschol.

[40] (2012). SAS Institute Inc.

[41] (2011). R Core Team.

[42] Hintze J (2011). NCSS, LLC.

[43] Raab GM, Butcher I (2001). Balance in cluster randomized trials. Stat Med.

[44] Turner EL, Li F, Gallis JA, Prague M, Murray DM (2017). Review of recent methodological developments in group-randomized trials: part 1-design. Am J Public Health.

