# Supplementary material for: Comparing the Effectiveness of Clinicians and Paraprofessionals to Reduce Disparities in Perinatal Depression via the Mothers and Babies Course: Protocol for a Cluster-Randomized Controlled Trial
Source: JMIR Res Protoc. 2018 Nov 20;7(11):e11624. doi: 10.2196/11624 (PMC6280028; doi:10.2196/11624)
Supplement: Multimedia Appendix 1 [file resprot_v7i11e11624_app1.pdf]

PATIENT-CENTERED OUTCOMES RESEARCH INSTITUTE
SUMMARY STATEMENT
(Privileged Communication)

**Principal Investigator:**	Darius Tandon
**Organization:**	Northwestern University
**Project Title:**	Comparing the Effectiveness of Clinicians and Paraprofessionals to Reduce Disparities in Perinatal Depression
**PCORI Funding Announcement:**	Addressing Disparities
**Review Cycle:**	2015: Cycle 2
**Request ID:**	2015C2-1507-31473

AVERAGE OVERALL SCORE: 25

QUARTILE: 1

In-Person Review Discussion Notes:
Strengths:

- The panel was enthusiastic about this study's potential to improve outcomes for low-income ethnic minority women suffering from perinatal depression.
- The research team has the appropriate expertise and experience working with the study population, and demonstrated strong likelihood of success in recruiting and retaining study participants.
- The comparators—Mothers and Babies (MB) intervention delivered by mental health professionals, MB delivered by paraprofessionals, and home visiting without MB—are well justified. Implementing a paraprofessional-led MB would reduce the major barrier of availability that currently limits the widespread adoption of MB. Widespread adoption of paraprofessional-led MB would align with the Affordable Care Act's priority of sending more health workers out to communities.
- The MB intervention is based on cognitive behavioral therapy, which has a strong evidence base, particularly when administered by mental health professionals.
- While MB delivered by paraprofessionals does not have widely reported demonstrated efficacy, investigators have provided sufficient preliminary data to justify conducting a CER trial for paraprofessional-led MB.
- The stated outcomes of reduction of postpartum depressive symptoms and improved quality of life, parenting practices, engagement in pleasant activities, and relationship with one's partner, are important to patients.

Weaknesses:

- While home visiting services are covered under the Affordable Care Act, the reduction in public health budgets in many states could mean that interventions based on home visiting services may not be sustainable.
- The study does not include children's data during the postpartum period to account for the negative effect of parental depression on their children.
- A moderate weakness is that the study does not address impact on women who are not already participating in home visiting or mental health services, such as those in rural and frontier states, where these services are very sparse.
- The investigators have not given sufficient attention to the co-learning aspect of stakeholder engagement because they have under-emphasized flow of information from stakeholders to investigators.
- Patients seem less engaged than stakeholders, which wasn't justified in the application; the role of patients in dissemination to promote change in practice should be clarified.

The following reviewer critiques were completed prior to the in-person review and were not altered post-

discussion.

Criterion 1: Impact of the condition on the health of individuals and populations

Reviewer 1:

Strengths:

- There is a major strength that the application proposes to compare three study conditions (two intervention conditions and usual care) aimed at reducing depressive symptoms and/or preventing major depressive disorder (MDD) in a vulnerable population. Information about prevalence, morbidity, loss of productivity and other negative maternal and infant outcomes associated with depressive symptoms is well presented.

Weaknesses:

- Some references cited in this section are not recent (e. g., 1996, 2001 etc.).

Reviewer 2:

Reviewer 3:

Reviewer 4:

Strengths:

- The proposal makes a strong case that postpartum depression (PPD) is a common and serious problem.
- The proposal also makes the case that PPD is particularly common in low-income women and others who are traditionally underserved.
- PPD has wide impact on both mothers and their families.

Weaknesses:

- None noted.

Criterion 2: Potential for the study to improve health care and outcomes

Reviewer 1:

Strengths:

- The proposed study focuses on a priority area identified by PCORI (i.e., interventions for improving perinatal outcomes), Illinois Governor's Office on Early Childhood Development, the target population, clinicians and researchers. The findings will address a critical gap in current knowledge with policy implications (e.g., resource identification and allocation). This is a major strength.
- The proposed study is innovative in comparing efficacy of MB delivered by paraprofessionals vs. usual care, and effectiveness of group-format MB intervention delivered by mental health professionals vs. paraprofessional home visitors.
- Findings of three randomized controlled trials suggest significant reductions in depressive symptoms and effects on prevention of new cases of major depression among intervention (both clinician and home visitor led) participants compared to usual care participants. The team has developed a thoughtful dissemination plan with support of state-level officials which will result in timely improvements in practice and patient outcomes if positive findings are found (major).

Weaknesses:

- Since depressive symptoms have direct and indirect impact on both mothers and their children's health and development, it would be beneficial to include children's data during the postpartum period to inform the potential effect of the intervention on children's short-term outcomes (minor).

Reviewer 2:

Strengths:

- A moderate strength is that addressing maternal depression has been identified by Federal agencies (Administration for Children & Families, 2006; Health Resources & Services Administration, 2013), and Home Visitation (HV) model developers (Prevent Child Abuse America, 2001), and HV researchers (Ammerman et al., 2010) as a much needed and highly impactful HV enhancement.
- A major strength of the proposal is that it is innovative. It proposes the delivery of mental health services outside the public mental health system, is lower cost to deliver, and addresses many challenges postpartum women face in accessing services that are related to reimbursement.
- A major strength is that the research team has engaged in prior research activities focused on delivering behavioral health services via HV programs that have shown to be effective in reducing symptoms of depression in postpartum women.

Weaknesses:

- A moderate weakness is that the applicant does not clearly demonstrate that the research questions address a critical gap in current knowledge as noted in systematic reviews, guideline development efforts, or previous research prioritizations. The application only includes general references to the study's fit into existing research portfolios without discussing a current gap in knowledge clearly.
- A minor weakness is that the applicant does not clearly demonstrate that patients have identified that research related to HV and the Mothers and Babies (MB) course is important overall.
- A moderate weakness is that the applicant does not clearly describe the potential study impact related to the number of perinatal women at risk for postpartum depression that do not engage in mental health services, or the percentage of these women estimated to be served through HV programs, as the study interventions will be focused on delivering mental health services through HV programs.

Reviewer 3:

Strengths:

- The proposed study is innovative and addresses a critical gap in knowledge in that it seeks to evaluate the effectiveness of the delivery of Mothers & Babies, an effective intervention to prevent postpartum depression, by paraprofessionals during home visits.
- The research has been identified as important to community members who collaborate with this team of researchers.
- The findings of this comparative effectiveness study would provide sizable benefits to the prevention of postpartum depression in low-income women.
- It is likely that the findings from this study could be disseminated and implemented quickly in practice. There is a strong plan for disseminating the findings of the study.

Weaknesses:

- None noted.

Reviewer 4:

Strengths:

- The proposal makes a strong case that it would be advantageous to learn whether paraprofessionals can deliver an equivalent intervention to professionals. This is a major strength.
- The proposal makes a strong case that the MB course, delivered by professionals, is effective. The proposal reports results from several randomized trials documenting the benefits of the intervention. This is a major strength.
- If intervention by paraprofessionals is equivalent to professional intervention, this evidence-based intervention could be more broadly introduced. This is a major strength.

Weaknesses:

- It is not clear how widespread home visits (HVs) are in other communities. If this is not a common practice then the ability to disseminate and implement the intervention would be limited. This is a moderate weakness.

Criterion 3: Technical merit

Reviewer 1:

Strengths:

- The applicant has adequately justified the choice of comparison intervention based on the input from stakeholders; the comparison interventions are realistic options in the current practice.
- The proposed sample size and power estimates are based on realistic and careful evaluations of the anticipated effect size.
- The proposed plan and strategies for sample recruitment, enrollment and retention are feasible and well justified based on the applicants' prior work.
- The applicant has proposed a rigorous study design in all aspects with realistic timeline and essential resources. The team members have the necessary expertise and experience to conduct the proposed research.
- The applicant has provided strong justification for the study population targeted in this proposed research.

Weaknesses:

- The applicant has not delineated a clear theoretical framework that illustrates relationship among intervention components, anticipated outcomes and measures (minor).
- The applicant has not fully described sample inclusion and exclusion criteria in the Methods section (e.g., significant cognitive limitation as an exclusion criterion is only mentioned in the "Protection of Human Subjects." How significant cognitive limitation is defined and measured is not mentioned either (minor).
- The applicant has proposed to measure participants' relationship with their partners. However, it is unclear whether existence of "partner/spouse/significant other" is one of the inclusion criteria for study participation (minor).
- Since the proposed study targets mothers with depressive symptoms and competing life priorities, it is important to consider participant burden of completing study activities, including the questionnaire. It would be helpful if the investigators addressed this concern in the proposal (minor).
- Page 15: please describe briefly what "generally accepted practices for analyzing qualitative data" is (minor).
- The intervention effect may be strengthened by meaningfully engaging participants' partners/family (minor).

Reviewer 2:

Reviewer 3:

Reviewer 4:

Strengths:

- The research design is strong and has been improved on revision. This is a randomized trial that compares usual care, intervention by professionals, and intervention by paraprofessionals. This is a major strength.
- The inclusion of a usual care arm strengthens the proposal by allowing the investigators to determine whether paraprofessionals can attain results that are better than usual care.
- The measures have been improved on revision. The change in depression measurement is well justified and improves the rigor of the project.
- The plan for recruitment appears feasible, given the extensive experience of the team in previous research with this population.

Weaknesses:

- There is relatively little evidence for the efficacy of the paraprofessional arm of the study. Thus it is unclear how well this trial fits the definition of a comparative effectiveness study. This represents a moderate weakness.

Criterion 4: Patient-centeredness

Reviewer 1:

Strengths:

-There is a major strength that the proposed research focuses on questions that affect outcomes of interests to the target population, caregivers/family, clinicians, researchers and policy makers (major).

-The proposed research addresses key questions mentioned in PCORI's definition of patient-centered outcomes research (major).

Weaknesses: none noted.

Reviewer 2:

Strengths:

- A moderate strength of the proposal is that the it includes letters of support from several state HV programs that clearly demonstrate that program staff are supportive of implementing group versions of the MB programs.

Weaknesses:

- A moderate weakness is that although stakeholder interest in the study topic is strong, patient interest in the topic of the MB program delivered in a group format is not described in detail in the proposal.
- A moderate weakness is that the applicant does not clearly describe how the proposed research addresses one or more of the key questions mentioned in PCORI's definition of PCOR, although the question focused on how clinicians and health care delivery systems can help patients make better decisions about care is indirectly addressed.

Reviewer 3:

Strengths:

- This proposal has demonstrated patient-centeredness in the development of the research question, the development of the proposal and the dissemination plan of the findings.
- It addresses the key question in PCORI's definition on what are the options for the prevention of postpartum depression in the community setting. Ethnically diverse and low-income women will benefit from the delivery of Mothers & Babies via home visits.

Weaknesses:

- None noted.

Reviewer 4:

Strengths:

- The proposal focuses on outcomes that are highly valued by stakeholders, based on the information presented in the proposal when these outcomes were evaluated by patients and families.
- The proposal addresses a major barrier to widespread application of a successful model. Although professionally administered depression intervention is effective, cost and availability of trained professionals has limited adaptation of the model. Using paraprofessionals, if proven successful, would diminish both of these barriers and make the program much more feasible to implement widely.
- The letters of support are strong and address every element of patient-centeredness.

Weaknesses:

- None identified.

Criterion 5: Patient and stakeholder engagement

Reviewer 1:

Strengths:

-The applicant has clearly described the process of engaging the target population and other stakeholders in revising the intervention, formulating research questions, defining target population, comparators and outcomes, implementing the research, evaluating the outcomes, and suggesting plans for dissemination (major strength).

- The roles of decision-making authority of all partners have been clearly stated based on their expertise and experience (major strength).

-The proposal has demonstrated the principles of reciprocal relationships, co-learning, partnership, trust, transparency, and honesty.

Weaknesses: none noted.

Reviewer 2:

Strengths:

- A major strength of the proposal is that the study topic focused on delivering the HB curriculum to address post-partum depression via group delivery of HP classes was initiated by stakeholders involved in the delivery of state Maternal Child Health HV programs.
- An Operations Team/Community Advisory Committee will be used to inform the study , and 9 of the 12 members are patients or stakeholders.

Weaknesses:

- A moderate weakness is that the involvement of patients in developing the initial research questions is not clearly demonstrated in the proposal, although the applicant states that HV program staff represent the opinions of patients served by the programs.
- A minor weakness is that the Operations Team/Community Advisory Committee members are all from Illinois, and it isn't clear if the group will be expanded to reflect the geography covered by the study.
- A moderate weakness is that significant decision making authority related to the study will rest with the Executive Committee, and patients are not members of this Committee.
- A minor weakness is that the relationship between the Executive Committee, Operations Team/Community Advisory Committee, and National Advisory Committee, and authority of each committee is not clearly described.
- A minor weakness is that the plan for reciprocal relationships for stakeholder and patient engagement is not clearly described. A consensus decision making model will be used for the Operations Team/Community Advisory Committee, but how decisions made by this committee will filter up/across to the Executive Committee and National Advisory Committee is not clearly described.
- A moderate weakness is that the description of co-learning for the study focuses on a description of education to be provided by the scientific research team members to the patient and stakeholder participants, and not vice versa.
- A minor weakness is that the proposal does not clearly describe how patients will be engaged in suggesting plans for dissemination of study outcomes to promote changes in practice.

Reviewer 3:

Strengths:

- There is evidence that patients' caregivers, patients and stakeholders were involved in the development of this proposal, will continue to be involved during the conduction of this study and will provide feedback in the dissemination phase. This engagement includes the training plans for the paraprofessionals in the delivery of the intervention.
- The proposal clearly articulates the reciprocal relationships, co-learning partnerships, trust, transparency and honesty.
- This study provided a clear description of the roles that stakeholders, patients and scientist.

Weaknesses:

- None noted.

Reviewer 4:

Strengths:

- The investigators have a successful history of engaging stakeholders and have detailed procedures to continue this.
- Inclusion of patient stakeholders who have benefitted from the intervention is a strength.
- The inclusion of support from public officials who are enthusiastic about the program is a unique strength.

Weaknesses:

- None noted.

Overall Comments

Reviewer 1:

There is an urgent need to screen and provide necessary resources to reduce depressive symptoms and prevent new cases of MDD in perinatal women. The proposed study addresses outcomes important to the target population, their family, clinicians, researchers and policy makers. The proposed design is rigorous with a realistic timeline and essential resources. It has assembled an excellent team and engaged stakeholders and other key players in the entire study process. Clarifications in some areas (e.g., theoretical framework, sample inclusion/exclusion criteria, participant burden etc.) are needed.

Reviewer 2:

The proposal's major strength is that it addresses maternal depression, which has been identified by Federal agencies and state staff engaged in Maternal Child Health Home Visitation programs as an issue that needs to be addressed. The study proposes to compare the effectiveness of delivering mental health services in a group format through HV programs, which is a low-cost method to provide mental health services and reduce postpartum depression. This is a major strength as is addresses many reimbursement/cost related barriers to services for these women. Another strength is that the key study team members have engaged in prior research on this topic.

There are many moderate and minor weaknesses to the proposal. A moderate weakness is that the applicant does not clearly demonstrate that the research questions address a critical gap in current knowledge. Another moderate weakness is that the proposal does not clearly demonstrate that the study focus is of eminent importance to patients. An additional weakness is that the potential impact of the study is not clearly quantified, as the project has a narrow focus of pregnant women served by HV programs. Patient engagement in all aspects of the study is also another moderate weakness. Stakeholder engagement in general is clear, but patient representation is less so.

Reviewer 3:

This is an interesting proposal aimed at comparing the effectiveness of clinicians and paraprofessionals to reduce disparities in perinatal depression. Postpartum depression is a serious mental health disorder that possesses a significant health and mental health risk for mothers and their infants, especially those of ethnically diverse and low-income backgrounds. Mothers and Babies is a group-based intervention that has been demonstrated to be efficacious in preventing the onset of major depression and reducing depressive symptoms when led by mental health clinicians. The specific aims of this proposal are: (1) To conduct a trial that compares the efficacy of Mother-Babies delivered by paraprofessional home visitors versus usual care (i.e., home visiting services without Mother-Babies); (2) to conduct a second trial that compares the effectiveness of Mother-Babies delivered by (a) mental health professionals versus (b) paraprofessional home visitors; (3) to determine whether effectiveness of the two versions of Mother-Babies (clinician led vs. home visitor led) varies according to patient characteristics (e.g., race, ethnicity, first-time mother) and/or geographic type of home visiting (HV) program (i.e., urban vs. rural), and (4) to examine the feasibility and acceptability of MB delivered by paraprofessional home visitors and mental health clinicians.

This proposal has several strengths, including the comparison on an already effective intervention to determine if the intervention can be delivered via home visits by trained paraprofessionals and a strong patient engagement plan. No weaknesses were noted at this time.

Reviewer 4:

This proposal expands on previous research that has shown that professional intervention included as a part of prenatal care can decrease rates of depression and depressive symptoms in pregnant women. The proposed project will compare a new intervention using paraprofessionals with the professional intervention, and contrast both with usual care. This is a strong proposal that is likely to produce valuable information on an important topic. The research

design and stakeholder engagement are strong. The authors have been very responsive to the feedback on the first round of reviews and have produced an impressive revision. There remains a concern about whether the paraprofessional arm of the proposed project can truly be described as an evidence-based intervention appropriate for a comparative effectiveness trial.

Does the application have acceptable risks and/or adequate protections for human subjects?

Reviewer 1: Yes

Most parts of the study protocol have acceptable risks and adequate protections for human subjects. Please clarify who is included in the “research team” for data access (p. 26).

Reviewer 2:

Reviewer 3: Yes

Reviewer 4: Yes
